# Probiotics in Alleviating Constipation: Mechanisms, Strain Screening, and Applications

**DOI:** 10.3390/biology15141212

**Published:** 2026-07-22

**Authors:** Hao Zhong, Huange Zhang, Muhammad Hussain, Tong Hu, Sixian Chan, Rongfa Guan, Jian Li, Ningxiang Yu

**Affiliations:** 1 College of Food Science and Technology, Zhejiang University of Technology, Hangzhou 310014, China; zhonghao@zjut.edu.cn (H.Z.); 211124260007@zjut.edu.cn (H.Z.); mhussainft11@gmail.com (M.H.); rongfaguan@163.com (R.G.); 2 Moganshan Institute ZJUT, Kangqian District, Deqing 313200, China; 3 College of Biotechnology and Bioengineering, Zhejiang University of Technology, Hangzhou 310014, China; hutong1992@zjut.edu.cn; 4 College of Computer Science, Zhejiang University of Technology, Hangzhou 310023, China; sxchan@zjut.edu.cn; 5 Key Laboratory of Geriatric Nutrition and Health, Beijing Technology and Business University, Ministry of Education, Beijing 100048, China

**Keywords:** probiotics, constipation, gut microbiota, SCFAs, 5-HT, screening strategies

## Abstract

Constipation is a prevalent gastrointestinal disorder with limited effective therapeutic options. Probiotics have emerged as promising microecological interventions, yet marked strain-specific efficacy and incomplete mechanistic understanding hinder clinical translation. This review systematically synthesizes evidence from human randomized controlled trials, animal studies, and mechanistic investigations on probiotic interventions for constipation. We catalog major anti-constipation strains across *Bifidobacterium*, *Lactobacillus*, *Bacillus*, and other genera, and summarize key findings from published meta-analyses indicating that *B. coagulans* improves stool consistency by 52.8%, *L. paracasei* demonstrates 86.3% effectiveness for incomplete defecation severity and 87.6% for PAC-SYM, while *L. reuteri* achieves 99.9% effectiveness on PAC-QoL. Mechanistically, probiotics act through four synergistic pathways: gut microbiota modulation, metabolite-mediated actions centered on SCFAs and 5-HT—with strains harboring the *abfA* gene cluster exhibiting enhanced arabinan utilization for sustained metabolite production—gut–brain axis neuro regulation, and dynamic mucosal barrier restoration. Finally, we propose a systematic four-stage screening pipeline integrating genomic pre-screening, probiotic property evaluation, in vitro functional prediction, and in vivo multi-tiered validation. This review provides a comprehensive framework for evidence-based strain selection and personalized probiotic therapy in constipation management.

## Simple Summary

Constipation is a common digestive problem that greatly affects quality of life. Probiotics offer a promising treatment option, but different bacterial strains vary widely in their effectiveness, making it difficult to choose the right one. This review systematically compiles current evidence on probiotic strains that relieve constipation, focusing on which strains work best, how they work through multiple mechanisms (including gut flora regulation, production of beneficial metabolites like SCFAs and serotonin, nervous system communication, and intestinal barrier repair), and how to efficiently screen for effective strains. This review provides a practical reference for clinicians, researchers, and industry professionals seeking evidence-based guidance on probiotic selection and personalized treatment strategies.

## 1. Introduction

Constipation, a frequently encountered digestive disorder, presents with reduced stool frequency, dry stool consistency, and difficult evacuation. Associated symptoms commonly include abdominal pain, appetite loss, fatigue and weakness. The incidence of functional constipation (FC) is between 10.1% and 15.3% worldwide, according to the Rome diagnostic criteria [1]. The prevalence of FC in the US, Canada and the UK is 7.6% [2], and the total prevalence in China is 8.5% [3], with female prevalence rates significantly exceeding male counterparts. The current treatment methods for constipation mainly focus on laxative treatment, such as taking laxatives or increasing fiber and water intake; however, up to 47% of patients report suboptimal satisfaction, primarily due to concerns regarding limited efficacy, safety risks, and adverse effects associated with these treatments [4,5].

Disruption of intestinal flora homeostasis has been established as an important inducement of various gastrointestinal diseases. Probiotics, owing to their potent antioxidant and anti-inflammatory activities, hold considerable promise for the prevention and management of such diseases [6,7]. A meta-analysis by Zhang et al. demonstrated that probiotic intervention, especially multi-strain combined intervention, can significantly reduce gut transit time (GTT), raise defecation frequency, and improve fecal consistency [8]. However, the therapeutic value of probiotics in constipation remains a subject of debate, and current national guidelines, along with most clinicians, do not advocate their routine use for this condition [9,10]. Although numerous studies have confirmed that probiotic interventions can effectively alleviate constipation symptoms without significant side effects, probiotics exhibit marked strain specificity, and the effective strains for constipation relief remain incompletely characterized. Moreover, the mechanisms of action and key molecular targets through which probiotic strains improve constipation are not yet fully elucidated, and the screening strategies for anti-constipation probiotics remain underdeveloped, making it difficult for clinicians and patients to make evidence-based strain selections. Based on this, this review synthesizes the full spectrum of evidence from strain selection strategies to mechanisms of action and preclinical validation. We systematically catalog the effective probiotic strains reported in the literature, elucidate their mechanisms of action and key functional targets, synthesize the screening strategies employed in previous studies, and finally discuss the translational applications of these probiotics in constipation management.

## 2. Constipation-Targeting Probiotic Species and Strains

Microecological therapeutics have been increasingly recognized as a promising strategy for treating intestinal disorders. It has been well documented in accumulating studies that constipation can be ameliorated by probiotics, which act to restructure the intestinal microbiota, facilitate bowel peristalsis, and raise the moisture level of stools. Nevertheless, due to the limited number and types of probiotics with anti-constipation effects reported at present, and the significant differences in their effect strains, the therapeutic efficacy of probiotics in constipation management remains contentious. In addition, distinct probiotic strains demonstrate differential therapeutic outcomes. A meta-analysis involving 3903 participants showed that the improvement rate of *Bacillus coagulans* on stool consistency was 52.8%; *L. paracei* was linked with the highest likelihood of alleviating incomplete defecation severity (86.3%) and also ranked highest in effectiveness on the PAC-SYM score (87.6%). For the PAC-QoL score, the highest probability of benefit was observed with *L. reuteri* (99.9%), followed by *B. bifidum* (59.0%) [11].

**Figure 1:**
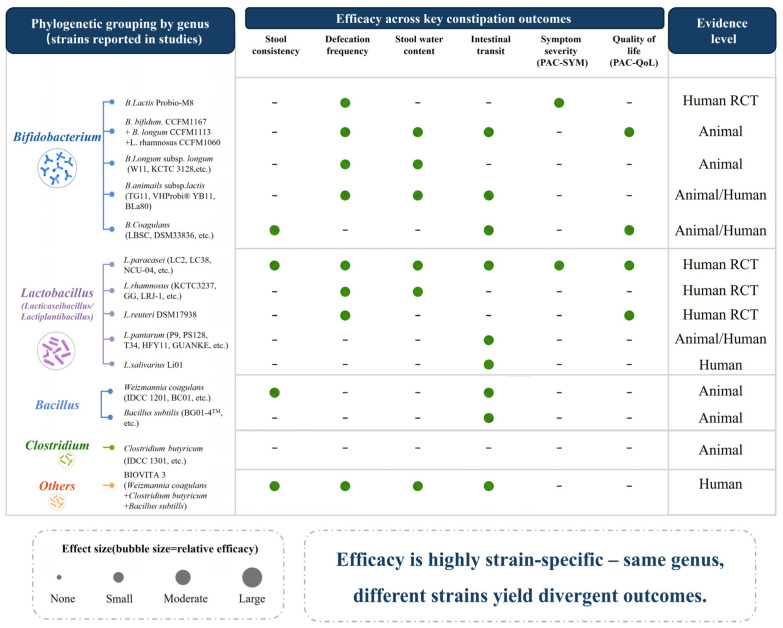
Strain-specific efficacy profiles of anti-constipation probiotics across key clinical outcomes.

Currently, the probiotics most frequently studied in the context of constipation management are *Bifidobacterium* and *Lactobacillus* species. Table 1 summarizes studies published in major databases including PubMed, Web of Science, and Scopus over recent years, focusing on effective probiotic strains with demonstrated anti-constipation effects, primarily from *Bifidobacterium* and *Lactobacillus*, along with other genera such as *Bacillus* and *Clostridium*. In the literature screening process, we prioritized English-language original research articles and high-quality reviews, while excluding conference abstracts, case reports, and studies beyond the immediate scope of this topic. Figure 1 visualizes the strain-specific efficacy profiles derived from meta-analyses and key clinical trials, highlighting the differential performance of individual strains across core outcome measures.

### 2.1. Bifidobacterium spp.

Among probiotics for relieving constipation, *Bifidobacterium* is the most frequently studied intervention measure for improving constipation and has been widely used as a microecological agent. Accumulated evidence identifies three principal *Bifidobacterium* species—*B. animalis*, *B. longum*, and *B. bifidum*—as key players in constipation alleviation through multimodal mechanisms [59,60]. Among them, *B. animalis* can enhance the function of the intestinal nervous system by triggering the release of neurotransmitters that regulate intestinal peristalsis, such as acetylcholine. It can compete with intestinal pathogens to maintain a healthy balance of intestinal microbiota, regulate intestinal immune response to facilitate the restoration of normal intestinal physiology, and finally alleviate constipation by softening stool and promoting defecation. Patients who routinely take its supplements had better bowel regularity and more normal stool consistency, according to long-term clinical research, indicating that it may be a dependable and long-lasting supplementary therapy option for constipation.

The improvement of bifidobacteria on constipation is not limited to a single strain, and its mixed strains seem to have a synergistic effect to further enhance the intervention effect. Most of the strains studied so far, the effective strains mainly include *B. lactis* Probio-M8 [12], *B. animalis* subsp. *lactis* TG11 [15], *B. animalis* subsp. *lactis* BLa80 [17], *B. animalis* F1-7 [25], *B. longum* subsp. *longum* CCFM1113 [13], *B. longum* subsp. *infantis* NKU FB3-14 [23], *B. lactis* TY-S01 [22], *B. longum* subsp. *longum* W11 [61], *B. longum* subsp. *longum* KCTC 3128 [14] and *B. bifidum* CCFM1167 [13], etc. Single or mixed use of the above strains can stimulate intestinal peristalsis and increase fecal moisture in different ways to ameliorate constipation. For instance, *B. animalis* subsp. *lactis* TG11 exerts a potent anti-constipation effect in loperamide-treated mice by regulating the level of gastrointestinal peptide hormone, up-regulating colonic mRNA expression of *Stem Cell Factor* (*SCF*) and *c-kit*, restoring the balance of intestinal flora, and regulating amino acid metabolism [15]. Similarly, *B. animalis* subsp. *lactis* BLa80 has the ability to modulate the level of gastrointestinal regulatory-related peptides, elevate the content of SCFAs, up-regulate the gene expression of gut-related proteins, successfully attenuate intestinal inflammation, and preserve the stability of the gut microbiota [17]. In addition, a 28-day randomized controlled trial (RCT) (*n* = 35) showed that *B. lactis* Probio-M8 strain can reach the intestine in a viable state to exert its probiotic effect, and can alleviate constipation symptoms by improving the type of defecation, increasing the frequency of defecation and reducing the intensity of defecation [12]. However, it is still important to note that the colonic dose-effect correlation of probiotics varies among strains, and the direct introduction of an overabundance of particular bacteria into the colon could upset the intestinal flora balance, consequently limiting their beneficial actions.

### 2.2. Lactobacillus spp.

In recent years, the number of *Lactobacillus* species persistently associated with the human gastrointestinal tract has been increasing, and they have been revealed to be closely related to numerous gastrointestinal diseases [62]. Focusing on constipation, *Lactobacillus* species have made certain contributions to its alleviation. Currently, the *Lactobacillus* species most studied in constipation intervention include *L. plantarum*, *L. paracasei*, *L*. *reuteri*, and *L*. *rhamnosus*. Some studies have also mentioned *Lactobacillus fermentum*, *L*. *gasseri*, and *L*. *salivarius*. They mainly exert their effects through the microbiota-gut–brain axis, enhancing intestinal peristalsis and increasing the water content in feces to achieve the effect of improving constipation.

Existing studies have shown that *L. plantarum* strains such as P9 [29,30], T34 [31], PS128 [32], NCU116 [63], YS-3 [64], and Guanke [34] have certain effects in improving constipation. Shen et al. conducted experiments on mice and zebrafish models and found that *L. plantarum* T34 exerts its anti-constipation effects in loperamide-induced models through a variety of mechanisms [31]. These include enhancing intestinal barrier function and restoring intestinal homeostasis, as well as regulating oxidative stress and the intestinal microbiota. In human trials, Liu performed a large-scale randomized, double-blind, placebo-controlled parallel trial, which showed that continuous intake of *L. plantarum* P9 for 28 days significantly relieved constipation symptoms in patients, and the therapeutic effect was maintained even after discontinuation of P9 [29]. It mainly achieves this by regulating the structure of the intestinal microbiota, increasing the abundance of beneficial bacteria and active metabolites, and providing energy for intestinal peristalsis through lysine degradation, phenylalanine metabolism, and various amino acid metabolic pathways. Additionally, studies have shown that *L. plantarum* BC299 in combination with black tea can effectively alleviate constipation in mice [28].

The therapeutic effects of *L. paracasei* strains such as NCU-04, FZJJH2L6, LC2, LC3, LC38, and FJSWX33-L2 on constipation have been verified through research. The pathways by which these strains alleviate constipation vary. *L. paracasei* LC2, LC38, and FJSWX33-L2 can all increase the content of 5-HT in the intestine to varying degrees [38]. LC2 can significantly increase the concentration of NT-3 in the colon [38]. *L. paracasei* FZJJH2L6 increases the content of propionic acid in feces by increasing the relative abundance of bacteria that produce SCFAs [40]. *L. paracasei* NCU-04 can alleviate constipation by regulating the levels of gastrointestinal peptides [39]. Moreover, compared to studies on single strains, many researchers seem to be particularly interested in the combined and synergistic effects of *L. paracasei* with other substances. Lu et al. used *L. paracasei* and konjac glucomannan as a compound laxative and verified its anti-constipation effect in a mouse model [65]. The results showed that it could alleviate constipation by increasing SCFA metabolism and the release of 5-HT. Zhao et al. explored the effect of the combination of β-1,3/α-1,3 glucan and probiotic powder on the defecation of constipated mice [35]. Hu Mingzhen studied the effect of *L. paracasei*-fermented wolfberry on constipation in mice [36]. The results of both studies indicated that the combination was effective.

Most studies on *L. reuteri* in the direction of constipation have focused on strain DSM17938. A meta-analysis that included five RCTs (*n* = 737) indicated that supplementation with *L. reuteri* 17938 can improve the defecation frequency of patients of all ages and has beneficial effects on patients with FC [43]. *L. rhamnosus* strains CCFM1068, FFJND15-L2, FZJHZ11-7, FHENJZ7-1 and FTJDJ11-1 can ameliorate loperamide-induced constipation via mechanisms that are independent of SCFAs. *L. rhamnosus* LRJ-1 has the therapeutic potential to relieve constipation by promoting the production of γ-aminobutyric acid (GABA) derived from intestinal symbiotic Bacteroides [42]. Additionally, *L. fermentum*, *L. gasseri* and *L. salivarius* are also useful for improving constipation. Stefan Roos et al.’s research indicates that oral supplementation of *L. gasseri* 345A can relieve the pain of FC and may improve intestinal emptying function [66]. Zhao et al. investigated the protective efficacy of *L. fermentum* strains derived from distinct phylogenetic lineages against loperamide-induced constipation in mice and concluded that *L. fermentum* YN54 significantly improved body weight, fecal moisture content, defecation efficiency, and gastrointestinal transit in the constipated mice [48]. *Ligilactobacillus salivarius* Li01 can effectively relieve constipation by regulating the 5-HT pathway and altering the intestinal microbiota, and can also synergize with the prebiotic psyllium husk to improve constipation in mice [49,50].

### 2.3. Other Strains

In addition to the aforementioned *Bifidobacterium* and *Lactobacillus*, some studies have also shown that certain species of *Bacillus*, *Clostridium*, and *Weissella* can improve constipation. These include *Bacillus subtilis* [67,68], *Clostridium butyricum* [56], and *Weissella cibaria* [58]. Research indicates that Bacillus subtilis can reverse intestinal motility dysfunction in mice with chronic constipation, mainly by promoting the release of 5-HT from enterochromaffin cells and regulating bile acid metabolism in the colon. Moreover, a double-blind randomized controlled trial (*n* = 67) has demonstrated that *Bacillus subtilis* BG01-4™ can alleviate specific symptoms of constipation and related gastrointestinal disorders in humans [51]. Zhou et al. investigated the laxative effect of *Bacillus coagulans* BC01 on mice with FC and found that it could effectively alleviate the adverse effects of constipation, with high-dose BC01 treatment exhibiting the greatest prophylactic efficacy against constipation [55]. Additionally, a probiotic powder mixture called BIOVITA 3, containing *Clostridium butyricum* IDCC 1301, *Weizmannia coagulans* IDCC 1201, and *Bacillus subtilis* IDCC 1101, has been proven to effectively improve constipation symptoms, significantly increasing the number of stools, stool moisture content, intestinal transit rate, and colonic mucosal layer [56].

### 2.4. Common Characteristics of Constipation-Targeting Probiotics

The improvement effect of probiotics on constipation is beyond doubt. However, the therapeutic effect of probiotics highly depends on strain specificity. This strain specificity seems to be related to certain specific functional genes, such as those involved in arabinose utilization, fatty acid hydratase, polysaccharide and cell membrane synthesis. Studies have shown that probiotics carrying specific functional gene clusters can regulate the metabolic capacity of the intestinal flora, shorten intestinal transit time and relieve constipation. Zhang et al.’s research indicates that *B. longum* with the *abfA* gene cluster can enhance the intestinal flora’s metabolic capacity for arabinan, promote the generation of SCFAs, and improve intestinal peristalsis function. Among them, the *abfA* gene cluster endows *B. longum* with a unique ability to utilize arabinan, providing glucose to the distal colon, promoting its adaptation and colonization in the host’s intestinal tract and generating beneficial metabolites [58]. Zhao et al. examined the effects of *L. fermentum* strains from various genetic backgrounds on loperamide-induced constipation in mice, and ultimately ascribed the strain-dependent differences to genes associated with fatty acid hydratase, polysaccharide production, and cell membrane synthesis [48].

In addition, most probiotics that exert anti-constipation effects can significantly increase the content of SCFAs after entering the gastrointestinal tract, promoting intestinal peristalsis by lowering intestinal pH, stimulating intestinal nerves and enhancing smooth muscle contractility. This indicates that most anti-constipation probiotics seem to be able to metabolize and produce SCFAs. The above-mentioned *B. longum* strain carrying the *abfA* cluster can improve FC in animals and humans by enhancing the utilization of arabinan, metabolizing to produce acetate and butyrate, which can improve intestinal peristalsis by interacting with G protein-coupled receptor 41 (GPR41) and G protein-coupled receptor 43 (GPR43) or directly acting on colonic smooth muscle [69,70]. It should be noted that Wang et al.’s study pointed out that the ability of *L. rhamnosus* strains to improve constipation symptoms is not related to the increase in SCFA levels in the colon [71].

## 3. Mechanistic Insights into Probiotic Interventions

**Figure 2:**
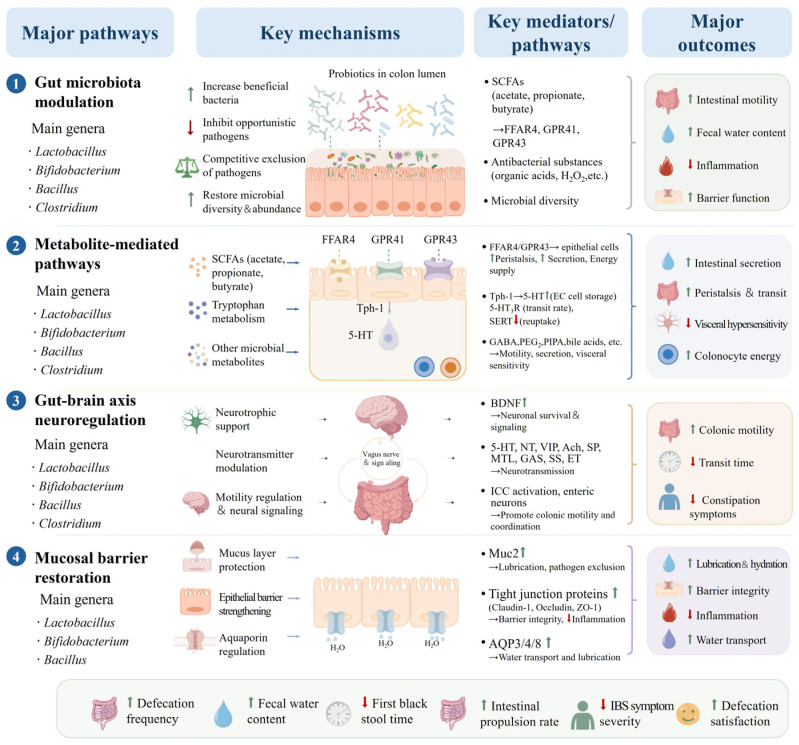
Multi-pathway mechanistic framework of probiotic action in constipation relief. “↑” denotes promotion or a positive effect, while “↓” denotes inhibition or a negative effect.

The pathogenesis of constipation is thought to be multifactorial, potentially involving dietary factors, metabolism, lifestyle habits, or other diseases. However, in general, the exact underlying mechanisms remain incompletely understood. As shown in Table 1, the therapeutic effects of probiotics on constipation have been verified in numerous human clinical trials and murine studies, but the relief of constipation varies, mainly including regulating the intestinal microecology, secretion of brain–gut peptides, and the immune system (Figure 2).

### 3.1. Gut Microbiota Modulation

The intestinal microbiota is a complex ecosystem of microorganisms living in the human gut, forming a symbiotic relationship with the host and participating in important physiological processes, having a significant impact on health. In recent years, numerous studies have shown that intestinal microecological imbalance can affect the intestinal environment and peristalsis, further promoting constipation. Specifically, it is manifested as a reduction in the number of dominant bacteria in the body and an increase in pathogenic bacteria. At the same time, the increased retention time of feces in the colon can lead to excessive reproduction of harmful bacteria, thereby aggravating the disorder of the intestinal microbiota. The two present a mutually reinforcing relationship. Secondly, microbiota imbalance can also lead to an increase in the expression level of the serotonin transporter (SERT) in the intestinal tract and a decrease in serotonin (5-HT), thereby altering intestinal motility, damaging the mucosal protective barrier, and further causing changes in intestinal motility and disruption of intestinal peristalsis, ultimately leading to constipation. Some studies have pointed out that the number of *Bifidobacterium* and *Lactobacillus* in the intestines of constipation patients decreases, while the number of some opportunistic pathogens increases [72]. Mancabelli et al. conducted a 16S rRNA-based microbial spectrum analysis on 147 fecal samples from 68 individuals with FC, and the data indicated that the numbers of *Bacteroides*, *Roseburia*, and *Faecalibacterium prausnitzii* in the intestinal microbiota of FC patients decreased [73]. In addition, the intestinal microbiota also affects intestinal motility. Abrams’ study on germ-free mice showed that, compared with wild-type mice, the gastric emptying and intestinal transit time increased in the absence of the gastrointestinal microbiota [74].

Probiotics can relieve constipation by regulating the microbiota balance, promoting the generation of SCFAs, and enhancing intestinal peristalsis. An increase in beneficial bacteria can, on the one hand, increase the content of antibacterial substances such as organic acids and hydrogen peroxide, and on the other hand, their metabolic products can compete with opportunistic pathogens for intestinal attachment sites, eliminate pathogenic bacteria, regulate the microbiota balance, and restore the intestinal barrier. Tang et al.’s study indicated that intervention with *B. lactis* TY-S01 in loperamide-induced constipated mice restored the intestinal microbiota abundance, increased the ratio of *Firmicutes* and *Bacteroidetes*, and Spearman correlation analysis demonstrated a significant association between metabolic product markers and the intestinal microbiota [22]. The above indicates that after TY-S01 restores the intestinal microecological balance, SCFA increase, and the two work together to prevent constipation. In clinical trials, the intake of *L. plantarum* can to some extent intervene in the composition of the intestinal microbiota of constipation patients, increase the abundance of beneficial bacteria such as *Akkermansia muciniphila*, *Bacteroides faecalis*, and *Lachnospiraceae*, and potential active metabolites, improve the stability of the intestinal microbiota, and thereby improve the clinical symptoms of patients and play a role in relieving constipation [30]. Notably, *Akkermansia* is increasingly recognized as a next-generation probiotic bacterium inversely associated with markers of intestinal inflammation [75].

### 3.2. Metabolite-Mediated Pathways

Probiotics can exert beneficial effects on human health by generating a series of beneficial metabolic products. Common metabolic products of probiotics include organic acids, antimicrobial substances, and neuroactive substances, among others. Some probiotics can also metabolize substances such as prostaglandin E2 in the body to influence constipation. These intestinal microbial metabolic products can affect the levels of protein peptides related to intestinal peristalsis, such as motilin, serotonin, and calcitonin gene-related peptide (CGRP), thereby influencing gastrointestinal peristalsis and transport function and causing constipation.

Neuroactive substances 5-HT metabolized by probiotics regulate intestinal secretion, peristalsis and vasodilation by activating multiple receptors. γ-Aminobutyric acid is also believed to improve constipation by regulating intestinal peristalsis and increasing fecal water content, making defecation smoother for patients [42]. From an apparent perspective, the genetically engineered probiotic strain EcN-5-HT that produces 5-HT can significantly improve intestinal peristalsis in a mouse model of constipation [76]. Liang’s research also found that *L. paracasei* FZJJH2L6 significantly increased the level of 5-HT in the intestine, resulting in a significant reduction in defecation time and an increase in fecal water content, which once again verified the key role of 5-HT in improving constipation [77]. 95% of 5-HT is produced by enterochromaffin cells (ECs) through multiple pathways, including transient receptor potential (TRP) ion channels (TRPV1/TRPV4), tryptophan hydroxylase (Tph), and olfactory receptor (OR) pathways [78]. Regarding the Tph pathway, most 5-HT is synthesized by ECs in the gastrointestinal mucosa through tryptophan hydroxylase-1 (Tph-1), and a small amount is synthesized through Tph-2 [79]. Tryptophan is converted to 5-hydroxytryptophan (5-HTP) under the action of Tph, and then to 5-HT through the action of decarboxylase and stored in the vesicles of ECs [80]. After 5-HT specifically binds to its receptors, it rapidly dissociates and is taken up into the cell by SERT and inactivated by monoamine oxidase, thus terminating its effect on the intestine. As a key and rate-limiting enzyme for 5-HT synthesis, changes in Tph levels affect 5-HT secretion by ECs [81]. Wei found that the induced loss of 5-HT production in Tph1-DTA mouse ECs did not cause changes in gastrointestinal morphology, but did lead to a decrease in in vitro intestinal motility and in vivo intestinal transit [82]. SERT is responsible for regulating the extracellular content of 5-HT; 5-HT can stimulate colonic motility through its receptors (5-HT_3_R and 5-HT_4_R), effectively increasing the gastrointestinal transit rate in multiple species [83]. Among them, 5-HT_4_R affects colonic anion and fluid secretion [84]; 5-HT_3_R has also been confirmed to be related to gastrointestinal peristalsis and can regulate the passage rate of food in the gastrointestinal tract [85]. At the same time, a prominent side effect of 5-HT_3_R antagonists is constipation [86]. Currently, *Bifidobacterium* has been proven to be a 5-HT_4_R agonist, which can enhance intestinal peristalsis and intestinal juice secretion, thereby alleviating constipation symptoms. Lu et al. found that after intervention with konjac glucomannan and *L. paracasei* X11, the expression levels of *5-HT_4_R* and *SERT* mRNA in mice returned to normal [65]. Meanwhile, Qiu et al. also found that compared with constipated mice, the levels of *5-HT* and *Tph1* mRNA in mice treated with *Ligilactobacillus salivarius* Li01 increased, indicating that different probiotics have different pathways of action on 5-HT [50]. In addition, as the main source of 5-HT, ECs can express voltage-gated ion channels and specific chemoreceptors that sense metabolites, stimulants, and catecholamines, and ECs can promote the release of 5-HT through voltage-gated calcium channels after activation [82]. Some studies have pointed out that *B. animalis* F1-7 can promote the expression of chromogranin A (CGA) and 5-HT and effectively relieve constipation, and free fatty acid receptor 4 protein (FFAR4) is a key target for 13,14-dihydro-15-keto-prostaglandin E2 to promote 5-HT secretion [87]. Among them, the metabolite 13,14-dihydro-15-keto-prostaglandin E2 of F1-7 has a potential improvement effect on constipation at a dose of 20 mg/kg body weight in vivo. In vitro, at a concentration of 30 ng/mL, it can effectively stimulate cells to secrete CGA and 5-HT, and also up-regulate the level of FFAR4. Lu et al.’s research found that *B. animalis* F1-7, *L. paracasei* F34-3 and *L. plantarum* FWDG can activate the CGA/α2A adrenergic receptor (ADRα2A) cascade signal and regulate the TRP/TPH-OR pathway to promote the secretion of 5-HT in ECs of constipated mice [88]. *Bacillus subtilis* can also significantly increase the release of 5-HT by ECs and activate the bile acid metabolism and its receptor TGR5/TRPA1 signaling pathway [67]. In addition to the above-mentioned mediating effects on the 5-HT generation pathway, *Clostridium* can regulate 5-HT signaling by producing soluble metabolites that affect 5-HT synthesis and influence gastrointestinal motility through this mechanism.

SCFAs, as one of the core metabolic products of probiotics, such as acetic acid, propionic acid, and butyric acid, can lower intestinal pH, activate intestinal mucosal receptors and the vagus nerve, and directly affect the peristalsis of colonic smooth muscle. Acetic acid can act on colonic mucosal cells, providing them with the main energy and promoting colonic peristalsis by enhancing electrolyte absorption; propionic acid can promote glycogen synthesis and provide energy for intestinal peristalsis; butyric acid mainly provides energy for colonic epithelial cells and affects the anti-inflammatory activity of epithelial cells. Butyrate, acetate, and propionate drive intestinal peristalsis by eliciting 5-HT release from enterochromaffin cells, with the released 5-HT subsequently activating 5-HT4 receptors on enteric neurons [89]. The translational significance of this pathway is supported by meta-analytic evidence showing that fecal propionate and butyrate are decreased in constipation-predominant IBS, while butyrate is increased in diarrhoea-predominant IBS [90]. Studies have shown that the content of SCFAs in the feces of constipated patients is significantly lower than that of healthy people [91]. *L. paracasei* can increase the content of propionic acid in feces by increasing the relative abundance of bacteria that produce SCFAs, and *L. rhamnosus* can increase the content of acetic acid and total organic acids in the feces of mice, both of which have certain improvement effects on constipation. In addition, after constipated patients consume *B.* CCFM16, the content of acetic acid and butyric acid in feces increases, and the frequency of defecation also increases. SCFAs can also promote the growth of beneficial bacteria and SCFA-producing bacteria, stimulate the production of mucin, and release peristaltic mediators such as 5-HT and CGRP, thereby regulating from multiple dimensions and alleviating the related symptoms of constipation. Furthermore, it is noteworthy that in the proximal colon, glucose supply is typically sufficient to meet the microbial requirements for the production of SCFAs, bile acids, and uracil. However, the production of SCFAs in the distal colon often declines due to inadequate glucose supply, thereby limiting their functional efficacy. Research indicates that strains of *Bifidobacterium longum* possessing key genetic determinants for arabinose utilization can stably proliferate and produce these beneficial metabolites, thereby enhancing the SCFA production pathway to alleviate FC [92].

The beneficial effects of SCFAs—particularly butyrate—on intestinal barrier function have been validated in multiple in vitro cell models, where they positively regulate the expression of claudin, zonulin, and occludin, leading to restoration of transepithelial electrical resistance. These effects are, however, dose-dependent: lower concentrations of butyrate confer barrier-enhancing benefits, whereas higher doses exert detrimental effects on Caco-2 monolayers [93]. In addition to its direct actions on epithelial integrity—which have also been confirmed in a murine model of barrier injury [94]—butyrate has been reported to modulate mucin production, though the available evidence is not entirely consistent. Specifically, butyrate regulates mucin 2, the dominant secretory mucin in the gut, either through direct epigenetic modifications (histone acetylation or methylation of the *MUC2* gene) or indirectly via fibroblast-mediated pathways, and butyrate enemas have been shown to increase mucin 2 synthesis in mice, albeit with a paradoxical decrease in mucosal layer thickness. Collectively, the net outcome of SCFA activity is reinforcement of the epithelial barrier, enhanced mucin secretion, and suppression of pro-inflammatory cytokine production. These effects are mediated through various G protein-coupled receptors (GPCRs) expressed on neutrophils, dendritic cells, macrophages, and T and B lymphocytes, thereby promoting an anti-inflammatory milieu within the intestinal mucosa [95].

Furthermore, probiotics produce bacteriocins, hydrogen peroxide and other antibacterial substances in vivo to inhibit the growth of pathogenic bacteria and restore the intestinal barrier, which has a certain impact on constipation. Some studies have also pointed out that the metabolic products of probiotics in vivo, such as bile acids, can activate the bile acid receptor (TGR5) on ECs to stimulate the release of 5-HT and activate TGR5 on intestinal neurons to stimulate the release of CGRP, both of which can promote peristalsis. In a recent study, pipecolic acid (PIPA) was identified as a non-volatile fecal metabolite and is considered a potential target mediator for constipation relief mediated by *L. casei* strain Shirota, and its effect on constipation also shows a positive and significant dose-effect relationship [96].

### 3.3. The Neuroregulatory System of the Gut–Brain Axis

The gut and brain are interconnected through multiple routes, including the enteric nervous system, the vagus nerve, and microbial metabolites. Gastrointestinal dysfunction, such as constipation, frequently accompanies neurological conditions, while persistent constipation has been linked to cognitive impairment. As a bidirectional communication system between the digestive tract and the central nervous system, the gut–brain axis, by governing the secretion of BDNF and gastrointestinal hormones, exerts control over intestinal contractions and peristalsis, which constitutes a key mechanism through which this axis contributes to constipation [97].

BDNF and neurotensin (NT), which were initially discovered in the brain, are not only believed to have an impact on the central nervous system, but are also considered to play a key role in the enteric nervous system [98]. BDNF may exert its effects by binding to the high-affinity receptor tyrosine kinase B or the low-affinity p75NT binding receptor, promoting neuronal survival and development, opposing neuronal apoptosis and enhancing neural signal transmission within the intestinal system [99]. NT acts as an endocrine regulator of the gut in peripheral neurons and has been proven to be a neurotransmitter of non-adrenergic non-cholinergic (NANC) excitatory nerves in the human digestive tract [100]. It can function through NANC nerves or directly act on colonic smooth muscle to affect colonic motility. Previous studies have shown that after patients received subcutaneous injections of BDNF and NT-3 at different frequencies for a period of time, BDNF accelerated the emptying of the proximal colon, and NT-3 accelerated overall colonic transport as well as gastric and small intestinal transport [101]. Both can stimulate intestinal peristalsis to varying degrees and increase the frequency of defecation in patients. Human experiments have also shown that responses mediated by adrenergic and cholinergic nerves predominate in the normal colon, while responses mediated by NANC nerves are more common in slow transit constipation colon [102].

Gastrointestinal hormones are small peptides, including brain–gut peptides such as 5-HT and vasoactive intestinal peptide (VIP). These hormones, which are distributed in both the digestive tract and the nervous system, have been identified as key mediators in the gut–brain axis, playing an essential role in regulating intestinal motility and the bidirectional communication between the brain and the gut. Numerous studies on constipation have indicated that the levels of substances such as somatostatin (SS) and endothelin-1 (ET-1) are higher in constipated patients than in healthy individuals, while the levels of motilin (MTL), gastrin (GAS), serum substance P (SP), and acetylcholine (Ach) are relatively lower [15]. These neurotransmitters and gastrointestinal hormones play different roles in the intestine. Probiotics can alleviate constipation by regulating hormone levels. Among them, GAS, SP, MTL, and Ach are excitatory regulatory substances, while SS and ET-1 are inhibitory regulatory substances. VIP is an important regulator that can maintain intestinal relaxation and regulate intestinal fluid secretion. Several researchers have observed that the level of VIP decreases when constipation is improved through intervention [103,104]. However, other studies have shown that the level of VIP in constipated mice increases after probiotic intervention, and constipation is effectively relieved [15,105]. Despite the inconsistent observations of VIP level changes in the above studies, VIP is generally considered to have a positive effect on relieving constipation. However, excess VIP has been shown to suppress colonic motility in STC [106]. GAS in the intestine is mainly responsible for promoting gastric acid release, increasing gastric antral muscle contraction and intestinal motility [107]; SP acts on interstitial cells of Cajal (ICC) and cholinergic nerves to release acetylcholine, causing strong intestinal smooth muscle contractions and enhancing the propulsive force of the colon segment [108,109]. It can also affect the TRP and Tph pathways [110]; MTL promotes gastrointestinal motility by promoting the release of pepsin and duodenal contractions; Ach is mainly responsible for regulating muscle contractions and mucus secretion, and can relieve constipation by increasing intestinal contractions and promoting defecation [111]; SS can inhibit the excitation of cholinergic nerves, reduce the release of acetylcholine, and also inhibit the release of gastrointestinal hormones, thereby slowing down intestinal transit time and worsening constipation [112,113]; ET can promote vascular contraction and enhance intestinal smooth muscle contractions to worsen constipation. Several neurotransmitter receptors have been identified in ICC, including some excitatory and inhibitory neurotransmitters such as SP and VIP. ICC can receive neurotransmitters through these receptors and then transfer them to adjacent smooth muscle cells through gap junctions [106]. Some researchers have also found that ICC responds to 5-HT released by the mucosa, which depolarizes the membrane potential and increases the frequency of pacemaker activity in colonic ICC [114]. In summary, ICC is an important site for gastrointestinal motility and intestinal smooth muscle contractions. It can transduce inputs from intestinal motor neurons and generate intrinsic electrical rhythms, thereby coordinating the rhythmic contractions and relaxations of the intestine and having a certain impact on constipation. Probiotics can regulate the levels of these neurotransmitters to a certain extent, promote intestinal motility, and alleviate related symptoms.

### 3.4. Dynamic Mucosal Barrier Restoration

The intestinal barrier consists of three main components: mucus secreted by epithelial cells, the intestinal epithelial cells themselves, and their intercellular tight junctions. This barrier is critically involved in the pathogenesis of various intestinal disorders. Among them, the mucus barrier is mainly composed of mucin secreted by goblet cells and numerous immune mediators, responsible for lubricating the intestine and preventing the invasion of pathogenic microorganisms [25]. The intestinal epithelial barrier is located beneath the mucus layer and consists of a single layer of epithelial cells and intercellular connections, which is crucial for the intestinal mucosal barrier [115]. The intestinal mucosal barrier has selective absorption and secretion functions and can resist harmful substances such as antigens, pro-inflammatory factors and pathogens. This barrier helps maintain normal intestinal permeability and internal homeostasis, and its integrity is directly related to the dynamic balance of intestinal water and the softening of feces; while the integrity and efficiency of the barrier depend on the level of inflammation, the body’s antioxidant capacity, the secretion of mucin and the role of aquaporins, etc.

Studies have shown that inflammation can damage the integrity of intestinal epithelial cells, and oxidative stress can damage the intestinal mucosa, leading to gastrointestinal motility disorders and thereby exacerbating constipation. Conversely, the disruption of the intestinal mucosal barrier can cause intestinal permeability disorders and the invasion of pathogenic antigens into the mucosal tissue, thereby activating local immune activities and inducing severe inflammatory responses [116]. Mucin, secreted by intestinal goblet cells, helps form a mucosal barrier in intestinal epithelial cells and promotes intestinal lubrication by preventing water loss [25]. However, dysbiosis, pathogenic overgrowth, and excessive intake of mucin can disrupt the intestinal barrier system [117]. Research indicates that compared to healthy individuals, constipated patients have reduced secretion of soluble mucin [118]. The intake of probiotics can effectively regulate the balance of the gut microbiome, reduce inflammation, and enhance the body’s antioxidant levels, thereby repairing the intestinal barrier and restoring its normal function. In a mouse model of constipation induced by loperamide, He et al. found that the levels of inflammatory factors TNF-α and IL-1β and AQP4 were significantly increased, while the expression of intestinal barrier-related genes (Mucin-1 and Mucin-3) in the colon tissue was decreased [37]. However, the fermented wolfberry juice by *L. paracasei* reversed these trends, alleviating constipation symptoms in mice. This study demonstrated that *L. paracasei*-fermented wolfberry juice can improve constipation in mice by inhibiting inflammation and repairing the intestinal barrier. Shen’s research also showed that *L. plantarum* T34 and *L. acidophilus* YJ5 upregulated the expression of the mucosal barrier genes, including Claudin-1, Occludin, Muc2, and Muc3. These findings suggest that both strains enhance intestinal barrier integrity and relieve constipation through upregulation of these barrier-associated genes [31,119]. Mu et al. pointed out that Lactobacillus plantarum KFY02 can reduce the MDA content in constipated mice and increase the expression of SOD, CAT, GSH-Px, and GSH, thereby alleviating constipation [113]. Additionally, a randomized, double-blind, placebo-controlled clinical trial conducted on constipated patients (defined according to the Rome IV criteria) also demonstrated that after 30 days of multi-strain probiotic intake, the decreasing trend of GSH-Px and GST activities in red blood cells was weakened, and the sensation of incomplete defecation in constipated patients was partially relieved by ≥25% [120]. Zhang et al. found that probiotic preparations mainly composed of Bifidobacterium mainly alleviate constipation by repairing the inflammatory response caused by constipation, promoting gastrointestinal motility, increasing fecal water content, and relieving constipation; while probiotic preparations mainly composed of Lactobacillus mainly restore the levels of serum neurotransmitters (MTL, SP, and VIP) and inflammatory factors (IL-1, IL-6, and IL-8), increase acetic acid content, thereby reducing inflammation levels, changing fecal properties, and enhancing gastrointestinal motility [121].

Aquaporins (AQP) are water transport proteins in the intestine, abundant in the intestinal mucosa, responsible for regulating the transport of water and glycerol between cells, maintaining the osmotic pressure balance of the mucosal barrier, and closely related to the GI transport rate [122]. Currently, 13 types of AQP have been discovered in humans, among which AQP1, AQP3, AQP4, AQP8, and AQP9 play key roles in the intestine. They can regulate the water content of feces and have a certain impact on constipation. AQP1 acts as an absorption channel for water transport between the gastrointestinal mucosa and blood flow [123]. AQP9 can lubricate the intestine by promoting the secretion of colonic mucosa, thereby promoting excretion [72]. AQP3 is mainly expressed in the mucosal epithelial cells of the colon tissue and is believed to affect water transport in the gastrointestinal tract [124]. Its overexpression can aggravate constipation symptoms. Studies have shown that after taking an AQP3 inhibitor for 1 h, the water content of feces increases by about 4 times, leading to severe diarrhea [125]. Conversely, the expression of AQP3 is also related to constipation. Zhang et al. studied the expression of AQP in the colon of constipated rats and found that the mRNA expression of *AQP3* and *AQP8* genes in the colon tissue of the model group significantly increased, and this situation was improved after intervention with *Bacillus coagulans* [126]. *Bifidobacterium animalis* F1-7F1-7 can also inhibit the expression of the AQP3 gene in mice, increase colonic ion secretion, increase fecal water content, and thereby improve the state of feces [25]. AQP4 is usually located in the colon epithelial cells and expressed in the basal outer membrane of crypt cells at the bottom of the intestinal crypts and the basal outer membrane of the colonic surface epithelial cells, participating in colonic fluid transport [123]. Wang et al. pointed out that the water content of feces in AQP4 gene knockout mice increased compared to wild-type mice [127]. However, other studies have shown that in AQP4-deficient mice, the water permeability of the proximal colon decreased, but that of the distal colon did not, and the water content of feces was not different from that of wild-type mice [123]. AQP8 is an important water transporter in the gastrointestinal tract. Although the knockout model of AQP8 has little effect on colonic fluid absorption and fecal dehydration, and the water content of mouse feces does not change much [128], in many studies, the relief of constipation in mice is usually accompanied by a decrease in the expression of AQP3, AQP4, and AQP8 and an increase in the expression of AQP9 [129]. This indicates that the role of a single AQP in water absorption and secretion in the small intestine and colon is limited, and usually multiple AQPs need to work together to better regulate water absorption and maintain the osmotic pressure balance of the mucosal barrier. Probiotics can regulate the expression of AQPs, improve fecal water content, and thereby alleviate constipation-related symptoms. Studies have found that *Lactobacillus plantarum* CQPC02 can up-regulate the expression of AQP9 and down-regulate the expression of AQP3 [129]; *Bifidobacterium longum* can inhibit the significant increase in the levels of AQP4 and AQP8 in the model group; some Bifidobacterium bifidum strains can significantly reduce the expression of AQP8, thereby regulating the secretion and absorption of intestinal fluid and alleviating constipation-related symptoms [130].

## 4. Screening Strategies for Anti-Constipation Probiotics

**Figure 3:**
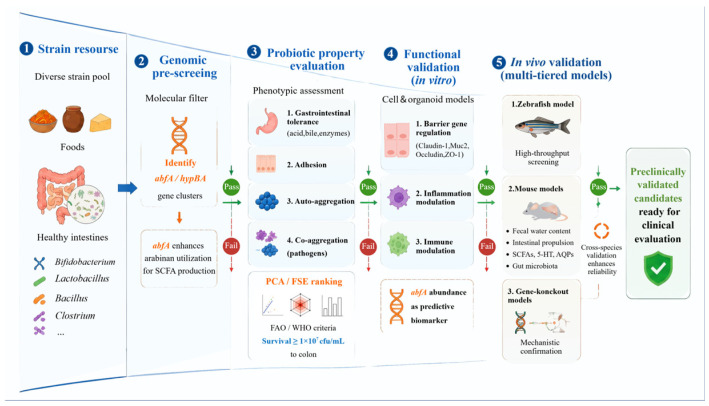
A screening pipeline for anti-constipation probiotics from strain resources to preclinical candidates.

The types of probiotics are diverse, but the types and quantities of probiotics with anti-colorectal-constipation effects are limited. Moreover, the therapeutic effects of probiotics on constipation often vary greatly among different strains within the same genus. Due to their elusive specificity, rational screening to effectively select probiotics for treating constipation remains a challenge for current research. Given their strain specificity, choosing effective strains in the prevention and improvement of constipation will help doctors and patients make personalized choices. Figure 3 outlines a screening pipeline from strain resources to preclinical candidates, incorporating genomic pre-screening (*abfA*/*hypBA*), probiotic property evaluation (PCA, principal component analysis/FSE, fuzzy synthesis evaluation), in vitro functional prediction, and three-tiered in vivo validation (zebrafish → mouse → gene-knockout).

### 4.1. Evaluation of Probiotic Properties

The gastrointestinal tract serves as the primary site of action for probiotic microorganisms. The premise underlying their potential health benefits is their capacity to survive transit through the digestive system, reaching the small or large intestine in sufficient viable numbers to adhere, colonize, and interact with the host. During this passage, however, probiotics are exposed to a series of hostile conditions, including the highly acidic gastric environment (pH 1.5–4.0), bile salts, and digestive enzymes. Therefore, it is usually necessary to ensure that the content of probiotics taken orally and surviving to the colon is greater than 1 × 10^7^ cfu/mL. The Food and Agriculture Organization and the World Health Organization have established a set of criteria for probiotic selection, which include tolerance to adverse physiological conditions, epithelial cell adhesion capacity, antimicrobial activity, and safety evaluation [131]. In current research, the evaluation of probiotics mainly includes GI tolerance, adhesion rate, self-aggregation ability, and the ability of pathogen and probiotic strains to co-aggregate, etc. Among them, GI tolerance is usually used as the initial step for evaluating probiotic strains, mainly to examine the survival ability of the strains under simulated gastrointestinal physiological conditions, in order to screen out strains with high acid resistance and good bile salt tolerance. Generally, the strains can be incubated in acidified media (such as MRS broth/buffer/peptone; pH 2.0–3.0, 1–4 h) or simulated gastric juice (with gastric protease present; pH 2.0–3.0) [132,133]. Moreover, more complex gastrointestinal models and dynamic models of human digestion are used to simulate various aspects of gastrointestinal transportation and evaluate the survival of probiotics during transportation, to provide a more comprehensive assessment of probiotic survival. These in vitro evaluation tests of acid and bile tolerance can usually serve as predictive indicators for the survival ability of strains in the host environment. Adhesion rate is the ability of probiotics to adhere to intestinal epithelial cells, which is a prerequisite for exerting the beneficial effects of probiotics. The ability to evaluate bacterial self-aggregation can screen out strains with relatively better colonization ability in the gastrointestinal tract. The analysis of the co-aggregation of pathogens and probiotics is a parameter reflecting the inhibitory effect of probiotics, which can evaluate the ability of probiotics to reduce pathogen levels through bacterial interaction. The evaluation of in vitro agglutination properties is helpful in screening out strains with excellent anti-pathogen ability. It is worth noting that for different functions, the probiotic properties to be examined are not exactly the same. For example, some probiotic strains can produce bile salt hydrolase, and the activity of probiotic bile salt hydrolase is considered an important indicator for selecting potential probiotic strains, as it is related to various mechanisms that help reduce the host’s plasma cholesterol levels [134]. Since each strain partially exhibits different probiotic properties in different aspects, some studies also use PCA and FSE to screen out the most promising strains. A study isolated 88 strains of lactic acid bacteria from traditional fermented foods. Among them, *L. plantarum* T34 was identified as having excellent probiotic potential through in vitro and in vivo screening. Maira et al. evaluated 30 thermophilic streptococcus strains by analyzing acid stress resistance, resistance to bile salts and oxidative stress, and then evaluated the bacterial adhesion ability using a mucin-producing HT29-MTX cell line, and used cell and strain co-incubation to evaluate the ability of the strains to regulate cytokine synthesis to regulate inflammatory responses [135]. Finally, PCA analysis clustered the strains into 6 categories. Liu et al. evaluated the acid and bile tolerance of 18 lactic acid bacteria and bifidobacteria strains using artificial gastric juice and 0.3% bile salts, thereby selecting *Bifidobacterium lactis* Probio-M8 and *Lactobacillus rhamnosus* Probio-M9 as excellent strains [136]. Shen et al. selected 10 strains from 30 strains by evaluating the tolerance, adhesion rate, in vitro agglutination characteristics, and antibacterial properties of the strains [137]. They then conducted a comprehensive assessment and ranking of the core probiotic characteristics of the 10 strains using PCA and FSE, and preliminarily identified the strains with potential for treating constipation.

### 4.2. In Vitro Screening of Probiotics for Constipation Relief

After ensuring that the probiotics can reach the gastrointestinal tract in sufficient quantities and exert effective effects, how to screen the probiotics that can effectively alleviate constipation remains a challenge. It is widely believed that subtle genomic variations usually lead to different functional characteristics of probiotics in the body. Cell and organoid models have been widely used to assess the regulation of key gene expressions related to the barrier function, signal transduction, inflammation, and differentiation of immune cells, which helps to predict the possible effects in complex organisms [138]. However, in terms of anti-constipation, due to the involvement of multiple systems and factors in a synergistic manner, cells and organoids are difficult to replicate this complexity and cannot quantify the relevant indicators of constipation. Currently, the feasible in vitro screening method in research is a proof-of-concept study based on the human cohort concept. The research team of Zhang et al. identified the key genetic factors of probiotics with constipation relief functions, and pointed out that the abundance of the *abfA* gene cluster in the intestinal microbiota can be used as a biological standard for predicting FC [92]. They first determined the core genes from 185 strains of Lactobacillus by pan-genomic analysis, and then conducted the prediction of gene CDS, identification of direct homologs in the genomes of effective and ineffective strains, as well as the identification and analysis of different functional genes or gene clusters in these genomes and their distribution in the strains. Finally, they determined the specific *abfA* and *hypBA* gene clusters. They pointed out that *Bifidobacterium longum* strains harboring these two gene clusters may represent a key determinant of probiotic anti-constipation potential. Subsequently, they further confirmed through animal studies that *B. longum* strains carrying the *abfA* gene cluster can supply glucose to the distal colon to support stable strain proliferation and the production of beneficial metabolites such as SCFAs, thereby enhancing the utilization of indigestible fibers in the intestine to improve constipation. This raises an intriguing question that remains to be addressed: whether other probiotic strains containing genes related to glucose supply in the distal colon also possess anti-constipation effects.

### 4.3. In Vivo Screening and Validation of Probiotics for Constipation Relief

When exploring the anti-constipation potential of probiotics, researchers employed various biological models to prove their effectiveness in vivo. Among them, the mouse model is currently the most widely used experimental system, followed by the zebrafish model. Both are often used as the initial screening or efficacy verification for probiotics for constipation relief. Among these models, zebrafish are commonly used for high-throughput screening, offering advantages such as high experimental efficiency and low cost, which make them particularly suitable for large-scale screening of interventions. Mice, on the other hand, are employed for mechanistic studies, given that their intestinal development, immune responses, and genetic makeup are highly similar to those of humans. At the same time, a large number of randomized controlled trials also conducted clinical research and validation using human patients with constipation as the research subjects. Although ideally, the final assessment of the probiotic’s function should be directly conducted in patients with constipation, considering the ethical limitations, high cost, and operability of clinical trials, the strains that can be included in clinical trials may need to undergo preliminary screening using appropriate in vivo models [139]. Currently, most of the evidence regarding the beneficial effects of probiotics on intestinal peristalsis mainly comes from studies based on mouse models [92]. Most studies verify their efficacy using mice after preliminary screening of probiotic characteristics, and some studies use animal models of different species for screening and validation. Shen et al. chose the zebrafish model as the in vivo screening model. After determining that *L. acidipiscis* YJ5 can promote intestinal peristalsis in zebrafish, they further verified the constipation relief ability using the mouse model, and the results were consistent with the former [119]. Additionally, their team also evaluated the efficacy of *Lactiplantibacillus plantarum* T34 with the zebrafish model and verified its anti-constipation potential using the mouse model [31]. It is worth noting that the judgment of the improvement effect of probiotics on the zebrafish model is highly dependent on the experimenter’s experience, as different researchers have different identifications of the time of the first peristaltic peak in the zebrafish intestine. Some researchers also used advanced feline and rodent knockout models when verifying the efficacy of probiotics. Zhu’s research showed that the composite probiotics can regulate the intestinal microbiota of kittens, improve constipation, and promote intestinal health. The research team of Zhang determined a key genetic factor responsible for effectively alleviating constipation in the animal model using ordinary mice, further verified its function using gene knockout experiments, and then conducted a nested case–control clinical trial and a human-mouse fecal microbiota transplantation experiment to further establish the functional role of *Bifidobacterium longum* in improving human constipation. It should be noted that the accuracy of the results obtained from animal models is not always verified in clinical trials.

## 5. Application of Probiotics for Constipation Treatment

The use of probiotics in the pharmaceutical and food industries is very widespread, as they can be administered in the form of drugs, foods, medical foods, dietary supplements, infant formula, or animal feed. Regarding probiotics for constipation, fermented dairy products remain the most popular form of probiotic consumption. Among them, Bifidobacterium has been added to dairy products as a probiotic for over 20 years, and fermented milk is also the most widely used and mature product for Bifidobacterium. Previously, numerous studies have shown that supplementing with probiotic yogurt or synbiotic yogurt can relieve constipation in humans and mice. Probiotic fermented milk containing *Lactobacillus paracasei* JY062 and *Lactobacillus gasseri* JM1, and fermented milk containing *Lactobacillus casei* Zhang and *Bifidobacterium animalis* ssp. lactis V9 can alleviate constipation symptoms by regulating the intestinal flora, inflammation, and metabolism [47,140]. Fermented soy milk by *Lactobacillus plantarum* CQPC01 and *Lactobacillus plantarum* CQPC02 can relieve constipation through their antioxidant effects [129,141]. Studies have shown that after consuming *Bifidobacterium yogurt* for three weeks, the frequency and quality of the subjects’ stools were significantly improved [142]. Meanwhile, a clinical trial involving 86 patients also demonstrated that yogurt intervention with multiple probiotics and prebiotics significantly improved constipation symptoms without showing serious adverse reactions [143]. In addition, other types of food containing probiotics, such as solid beverages, juices, and chocolate products, also occupy a small portion of the probiotic market and are available for consumers to consume. Zhang Liang and Yin et al.’s research indicates that probiotic composite solid beverages can regulate the intestinal flora to a certain extent and relieve constipation [144]. Probiotic chocolate containing *Streptococcus thermophilus* MG510 and *Lactobacillus plantarum* LRCC5193 (LYC) has a certain improvement effect on constipation induced by loperamide in rats [145]. The above studies all prove that probiotics have beneficial effects on relieving constipation. However, it should be noted that the effects of probiotics are species- and strain-specific, and more high-quality RCTs are needed to further demonstrate the effectiveness of these probiotic foods for constipation.

In clinical applications, an online survey of 1066 healthcare professionals from 30 countries/regions found that 79% of the respondents had recommended probiotics to their patients. Meanwhile, a survey of over 1500 primary care health professionals in the UK found that 78% of general practitioners recommended probiotics to their patients. Interestingly, a survey of 411 general practitioners and 365 gastroenterology experts revealed that 66% of general practitioners and 74% of gastroenterology experts did not recommend probiotics for treating constipation. The reason might be that various probiotic species and strains may have beneficial effects on intestinal motility and constipation in animal studies, but the data available for human studies are still limited, making it difficult to infer which probiotic strains may be the most clinically effective. The degree to which doctors agree with the scientific evidence for probiotics may influence their views on the effects of probiotics on symptoms and their behavior when recommending such products to patients. Interestingly, the primary channels through which the public obtains information about complementary and alternative therapies—including probiotics—for gastrointestinal conditions are family members, print media (newspapers and magazines), online platforms, and social circles. Consequently, the selection of probiotic products by both laypersons and clinicians may not always align with the prevailing scientific literature; rather, it could be shaped by product accessibility and marketing efforts.

**Figure 4:**
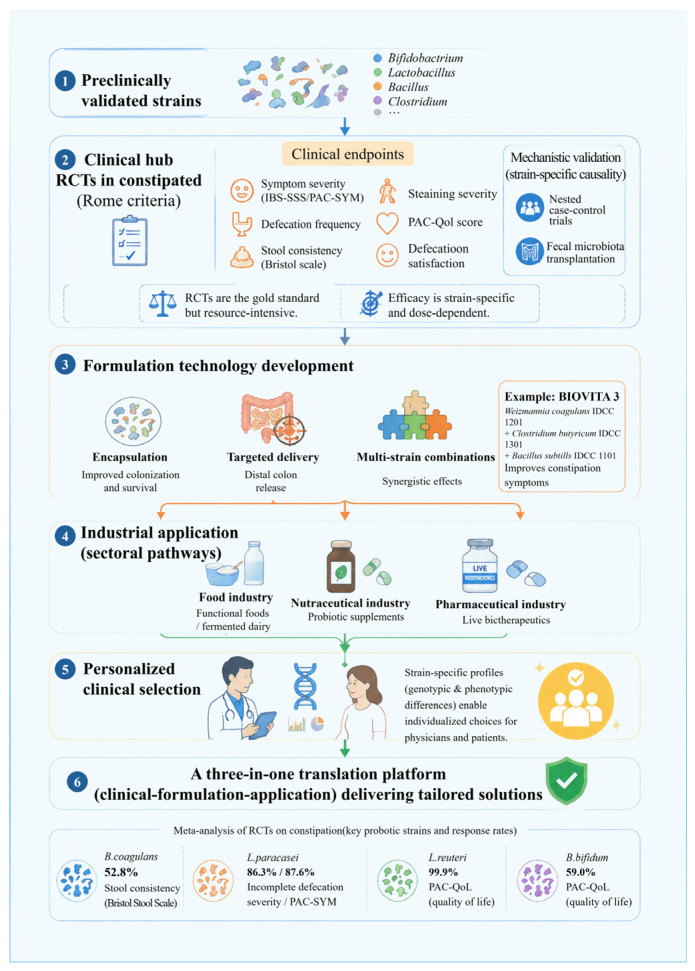
Translational application pathway from clinical validation to personalized healthcare solutions for anti-constipation probiotics.

To systematically address this translational gap between preclinical evidence and clinical application, Figure 4 presents a conceptual framework outlining the multi-stage pathway from clinical validation toward personalized healthcare solutions. Following rigorous RCTs that confirm strain-specific and dose dependent efficacy in constipated patients—with outcomes assessed using validated tools such as the Rome IV criteria for diagnosis, the Bristol Stool Scale for stool form, PAC SYM for symptom severity, and PAC QoL for quality of life, which together capture different yet complementary dimensions of constipation—three parallel formulation strategies (encapsulation for enhanced colonization, targeted delivery for distal colonic release, and multi strain combinations for synergistic effects) may facilitate the translation of effective strains into diverse product formats spanning the food (fermented dairy, beverages, chocolate), nutraceutical (supplements, solid beverages), and pharmaceutical (live biotherapeutics) sectors. We cautiously suggest that a more unified evaluation protocol could be considered in future research, perhaps comprising the Bristol Stool Scale, PAC SYM, PAC QoL, and weekly spontaneous bowel movement frequency as core outcome measures, while recognizing that further discussion on endpoint selection is warranted. Ultimately, these application channels are expected to converge on personalized clinical selection, where strain-specific genotypic and phenotypic profiles—particularly the *abfA* gene cluster as a candidate biomarker for sustained SCFA production—could in the future empower both physicians and patients to make more individualized therapeutic decisions, although this personalized approach remains an emerging area that requires further clinical validation.

## 6. Conclusions

Some strains of *Bifidobacterium*, *Lactobacillus*, *Bacillus*, *Clostridium* and *Weissella* have shown good effects in relieving constipation. Their mechanisms of action mainly include regulating intestinal balance, producing metabolic products, regulating the enteric nervous system, dynamically repairing the mucosal barrier and regulating aquaporins. Most probiotics can regulate the intestinal microecology to maintain balance and produce a series of beneficial metabolic products as long as the intake is sufficient. 5-HT is an important substance in the mechanism of relieving constipation. The targets of Bifidobacterium and Lactobacillus in relieving constipation focus on the expression of 5-HT_4_R, SERT, CGA, FFAR4 and ADRα2A. Different from *Bifidobacterium*, *Lactobacillus* can also affect the expression of *Tph1* mRNA. Bacillus subtilis can affect the expression of 5-HT by influencing the release of 5-HT by ECs and the TGR5/TRPA1 pathway. Clostridium mainly regulates gastrointestinal motility by affecting the soluble metabolites of 5-HT. Previous studies have mostly screened probiotics based on the improvement of symptoms, but probiotics have species and strain specificity, so it is difficult for us to determine which strain can effectively relieve constipation, which limits the application of constipation probiotics in the pharmaceutical industry, food industry and clinical practice. Future research should focus on screening constipation probiotics from the genetic level on the basis of ensuring that probiotics can reach the gastrointestinal tract in sufficient quantities and play their normal role, including genes related to the synthesis and expression of 5-HT, metabolic products and AQPs, and further screen and verify the dose-effect relationship of effective probiotics through animal and clinical trials, and finally determine the clinical safety and efficacy of these strains. Through methods such as embedding, the colonization rate of constipation probiotics in the gastrointestinal tract can be increased and applied in the food, pharmaceutical or clinical industries, etc., to facilitate consumers’ better choices.

## Figures and Tables

**Table 1:** Anti-constipation probiotic strains and their reported efficacy in human and animal studies.

Strain	Subject	Judgment Criteria	Intervention Dose and Time	Outcomes	Reference
*Bifidobacterium lactis* Probio-M8	human aged 20–50	Rome IV	1 × 10^10^ CFU/day, 28 days	increased frequency of bowel movements, improved defecation pattern, reduced bowel exertion	[12]
*B. bifidum* CCFM1167; *B. longum* subsp. *longum* CCFM1113; *L. rhamnosus* CCFM1060	C57BL/6 mice	/	8.75 × 10^9^ CFU/day, 4 weeks	enhanced stool water content, shortened time to first black stool, increased intestinal propulsion rate, improved colon tissue injury	[13]
*B. longum* subsp. *longum* KCTC 3128; *L. rhamnosus* KCTC 3237	zebrafish	/	/	increased intestinal contraction; enhanced gastrointestinal motility; alleviated inflammatory reactions	[14]
*B. animalis* subsp. *lactis* TG11	mice	/	10^6^, 10^7^ and 10^8^ CFU/kg, 16 days	promoted gastrointestinal motility; sustained fecal moisture; modulated gut microbiota composition	[15]
*B. animalis* subsp. *lactis* XLTG11	human aged 0–6	Rome IV	1 × 10^10^ CFU/day, 28 days	enhanced defecation frequency, increased beneficial bacteria, modulated genes involved in SCFAs and methane metabolism	[16]
*B. animalis* subsp. *lactis* BLa80	Balb/c mice	/	2.5 × 10^7^, 5 × 10^7^ and 1 × 10^8^ CFU, 15 days	facilitated intestinal peristalsis; retained fecal water; protected against gut barrier damage; and boosted SCFA production	[17]
*B. animalis* subsp. *lactis* BLa80	human aged 18–70	Rome IV	4–12 weeks	increased frequency of defecation, reduced IBS symptom severity, tended to normalise the type of stools, dropped the proportion of constipated patients	[18]
*B. lactis* Probio-M8 + *Lactobacillus rhamnosus* Probio-M9	sows	/	1 × 10^11^ CFU/g, 36 days	regulated the levels of inflammatory factors, increased in some potentially beneficial bacteria	[19]
*B. lactis* DSM 33812 and DSM 34614, *B. coagulans* DSM 33836	human aged 18–70	Rome IV	2 × 10^9^ or 5 × 10^9^ cells/day, 4/8/12/16 weeks	elevated the rate of patients with normalized stool form, decreased the number of patients with constipation, decreased pain occurrence and intensity, and increased bowel satisfaction	[20]
*B. longum* FGSZY6M4; *B. longum* FJSWXJ10M2; *B. longum* FSDJN6M3	SD rats	/	10^4^ and 10^8^ CFU, 28 days	modulated the levels of bioactive gastrointestinal peptides, altered the expression of 5-HT and its receptor; maintained fecal moisture	[21]
*B. lactis* TY-S01	Balb/c mice	/	10^9^ CFU/kg, 17 days	accelerated intestinal peristalsis, maintained fecal humidity, prevented gut barrier damage and intestinal dysfunction, repressed inflammatory responses, modulated the levels of key intestinal metabolites (SCFAs, 5-HT, enteric hormones)	[22]
*B. longum* subsp. *infantis* NKU FB3-14	Balb/c mice	/	2 × 10^8^ CFU/day, 4 weeks	modulated the expression of regulatory peptides and 5-HT, facilitated repair of gut barrier integrity, regulated the gut microbial community structure, increased SCFA content and elevated butyric acid abundance in feces	[23]
*B. animalis* subsp. *lactis* VHProbi^®^ YB11	Balb/c mice	/	10^9^ CFU/mL, 21 days	increased the fecal water content, modulated peristalsis-related neuropeptide concentrations, stimulated the intestinal peristalsis function	[24]
konjac glucomannan + *B. animalis* F1-7	KM mice	/	2 weeks	elevated stool moisture levels, accelerated the first black stool excretion, elevated small bowel transport rate, regulated the expression of gastrointestinal regulatory peptides	[25]
*B. bifidum* CCFM1163	C57BL/6 mice	/	1 × 10^9^ CFU/day, 21 days	enhanced the brain-derived neurotrophic factor; elevated SCFA levels; restored Cajal smooth muscle cells; improved intestinal permeability	[26]
*Lactiplantibacillus plantarum* AHQ-14, *Lactiplantibacillus plantarum* HM22, *Lactiplantibacillus plantarum* Y12	Balb/c mice	/	2 × 10^8^ CFU/day, 14 days	elevated stool moisture levels, restored colonic damage, enriched the populations of microbes linked to SCFA synthesis and AQPs expression, regulated the levels of gastrointestinal peptides	[27]
*Dark tea* + *Lactobacillus plantarum* BC299, *Dark tea* + *commercially available probiotic*	mice	/	10^9^ CFU, 21 days	increased fecal moisture, elevated small bowel transport rate, regulated the level of serum gastrointestinal peptide, regulated intestinal flora and relieved inflammation	[28]
*L. plantarum* P9	human aged 18–66	Rome IV	1 × 10^11^ CFU/day, 42 days	raised stool frequency, modulated gut microbial composition, adjusted the overall balance of intestinal, provided energy to the intestinal peristalsis through lysine degradation, phenylalanine metabolism, and amino acid metabolism pathways	[29,30]
*L. plantarum* T34	Balb/c mice	/	1 × 10^8^ CFU/day, 17 days	enhanced intestinal barrier function, restored gut homeostasis, promoted the production of 5-HT, maintained stability of water transport, raised fecal hydration	[31]
*L. plantarum* PS128	ICR mice	/	2 × 10^9^ CFU/day, 15 days	promoted intestinal motility, promoted mucin production, promoted serotonin signal transduction	[32]
*L. plantarum* HFY11	ICR mice	/	10^7^ and 10^8^ CFU, 12 days	accelerated the elimination time of the first batch of black stools, increased serum levels of gastrointestinal regulatory peptides, controlled the gut microbiota	[33]
*L. plantarum* GUANKE	ICR mice	/	3 × 10^9^ CFU/day, 22 days	alleviated composite constipation parameters (evacuation time, stool volume, moisture, and gastrointestinal transit), normalized the levels of intestinal factors, regulated the neurotransmitters and hormones, decreased levels of VIP and NO in serum	[34]
*β-1,3/α-1,3-glucan* + *L. paracasei*, *β-1,3/α-1,3-glucan* + *L. rhamnosus*, *β-1,3/α-1,3-glucan* + *B. latisticus*	KM mice	/	5 × 10^11^ CFU, 14 days	reduced the latency to first black stool, raised stool weight and count, elevated fecal water content, promoted intestinal peristalsis	[35]
*L. paracasei* NXU-19004 fermented goji berry juice	Balb/c mice	/	3 weeks	increased the number of stools, fecal moisture and propulsion rate, reduced the damaged integrity of the ileal wall, decreased the secretion of pro-inflammatory factors, regulated AQP levels	[36,37]
*L. paracasei* LC2, *L. paracasei* LC3, *L. paracasei* LC38, *L. paracasei* FJSWX33-L2	Balb/c mice	/	10^9^ CFU/day, 30 days	regulated the level of peptide tyrosine-tyrosine, neurotrophin-3, 5-HT, AQP, SCFAs, shortened time to first black stool, increased stool moisture	[38]
*L. paracasei* NCU-04	Balb/c mice	/	10^8^ and 10^9^ CFU/day, 20 days	regulated neurotransmitters, promoted GI motility and lubrication, maintained gut microbiota homeostasis	[39]
*Lactobacillus paracasei* FZJJH2L6	C57BL/6 mice	/	1 × 10^9^ CFU/day, 2 weeks	enhanced colonic neurotransmitter activity, increased mucus secretion, mitigated the inflammatory response, maintained intestinal Micro-ecological balance, increased propionic acid content	[40]
*L. rhamnosus* VHProbi^®^M15	Balb/c mice	/	1 × 10^9^ CFU/day, 21 days	increased fecal water content, improved fecal SCFA levels, regulated serum levels of gastrointestinal regulatory-related peptides, restored intestinal flora	[41]
*L. rhamnosus* LRJ-1, *B. uniformis* ATCC 8492	Balb/c mice	/	5 × 10^8^ CFU/day, 14 days	increased stool frequency, elevated stool moisture levels, restored colonic damage,	[42]
*Lactobacillus reuteri* DSM 17938	Five high-quality RCTs involving 737 participants	/	/	increased bowel movements	[43]
*Lactobacillus reuteri* DSM 17938 + MgO	human aged 6 months to 6 years	Rome IV	2 × 10^8^ CFU/day, 4 weeks	raised bowel movement frequency	[44]
Probiotic fermented milk (*Lactobacillus* MWLp-12 and *Lactobacillus* MWLf-4)	Balb/c mice	/	10 mg/kg·bw, 15 days	elevated fecal moisture, accelerated the first black stool excretion, enhanced small intestinal propulsion, increased concentrations of SCFAs, regulated the levels of gastrointestinal peptides	[45]
*Lacticaseibacillus rhamnosus* IDCC 3201	human	Rome IV	1 × 10^10^ CFU, 8 weeks	improved straining and constipation-related discomfort, enhanced bowel-habit satisfaction, enriched beneficial microbes and metabolites	[46]
Probiotic fermented milk (*Lacticaseibacillus parasei* JY062 and *Lactobacillus gasseri* JM1)	KM mice	/	10 mg/kg·bw, 14 days	enriched beneficial bacteria, raised fecal SCFA content, expanded the population of ICC, upregulated the expression of c-kit and SCF	[47]
*L. fermentum* YN54	Balb/c mice	/	5 × 10^9^ CFU/day, 17 days	elevated stool moisture, stimulated peristaltic activity, facilitated small intestinal transport, regulated gastrointestinal peptides, elevated SCFA content	[48]
Psyllium husk + *Ligilactobacillus salivarius* Li01	C57BL/6 mice	/	5 × 10^9^ CFU/day, 14 days	improved fecal water content, promoted defecation frequency, improved intestinal propulsion efficiency, modulated the hormonal milieu of the GI tract	[49]
*Ligilactobacillus salivarius* Li01	C57BL/6 mice	/	4 × 10^9^ CFU/day, 15 days	increased gut motility, improved intestinal fluid secretion, modulated serum concentrations of gut-regulatory peptides, regulated 5-HT pathway	[50]
*Bacillus Subtilis* BG01-4^TM^	human aged 18–75	Rome IV	5 × 10^9^ CFU/day, 4 weeks	improved the symptoms of constipation	[51]
*Lactulose* + *Saccharomyces boulardii*, *Lactulose* + *Bacillus licheniformis*	ICR mice	/	6 × 10^8^, 8 × 10^8^, 2 × 10^9^ and 3 × 10^8^ CFU/kg/d, 2 weeks	improved stool number and water content, increased total SCFA content	[52]
*Saccharomyces boulardii*	C57BL/7 mice	/	1.92 × 10^7^ CFU/kg·bw, 14 days	up-regulated key proteins affecting water reabsorption and inflammation, regulated small intestinal metabolite levels	[53]
*Bacillus coagulans* LBSC	human	Rome IV	6 × 10^9^ spores/day, 35 days	improved degree of stool consistency and stool expulsion	[54]
*Bacillus coagulans* BC01	KM mice	/	4 × 10^8^ and 4 × 10^9^ CFU/day, 11 days	elevated fecal moisture, stimulated peristalsis, regulated the secretion levels of excitatory gastrointestinal hormones, downregulated inflammatory proteins, regulated the homeostasis of the intestinal microbiota	[55]
BIOVITA 3 (*Weizmannia coagulans* IDCC 1201 + *Clostridium butyricum* IDCC 1301 + *Bacillus subtills* IDCC 1101)	SD rats	/	1 × 10^9^ CFU/day, 14 days	increased fecal output and moisture, elevated the gastrointestinal transit ratio, thickened the colonic mucosal layer, altered the gut microbiome, increased total SCFAs, promoted bowel regularity, maintained intestinal barrier integrity, regulated immune function	[56]
*Weizmannia coagulans* BC99	Balb/c mice	/	2 × 10^7^, 2 × 10^8^ and 2 × 10^9^ CFU/day, 14 days	elevated fecal moisture and gastrointestinal transit, increased gut microbial metabolism and butyrate generation, inhibited inflammation reaction, modulated gastrointestinal regulatory peptide levels	[57]
*Weizmannia coagulans* BC99	human aged ≥20	Rome III	2 × 10^9^ CFU/day, 8 weeks	alleviated constipation symptoms and enhanced quality of life in constipated adults, increased the levels of neurotransmitters, improved levels of anti-inflammatory factors	[58]

## Data Availability

No new data were created or analyzed in this study. Data sharing is not applicable to this article.

## Abbreviations

The following abbreviations are used in this manuscript:

SCFA	Short-chain Fatty Acid
5-HT	5-Hydroxytryptamine
FC	Functional Constipation
GTT	Gut Transit Time
SCF	Stem Cell Factor
RCT	Randomized Controlled Trial
GPR41/43	G Protein-Coupled Receptor 41/43
SERT	Serotonin Transporter
CGRP	Calcitonin Gene-Related Peptide
EC	Enterochromaffin Cell
TRP	Transient Receptor Potential
TPH	Tryptophan Hydroxylase
OR	Olfactory Receptor
5-HTP	5-Hydroxytryptophan
TGR5	Takeda G protein-coupled receptor 5
BDNF	Brain-Derived Neurotrophic Factor
NT	Neurotensin
NANC	Non-Adrenergic Non-Cholinergic
VIP	Vasoactive Intestinal Peptide
SS	Somatostatin
ET-1	Endothelin-1
MTL	Motilin
GAS	Gastrin
SP	Substance P
Ach	Acetylcholine
ICC	Interstitial Cells of Cajal
AQP	Aquaporin
PCA	Principal Component Analysis
FSE	Fuzzy Synthesis Evaluation
